# Some Causes that Lead to Invalidism in Women

**Published:** 1894-11

**Authors:** J. B. S. Holmes

**Affiliations:** Atlanta, Ga.; Adjunct Professor of Obstetrics and Gynecology, Southern Medical College, Atlanta, Ga.; Vice-President of the Southern Surgical and Gynecological Association; President of the Tri-State Medical Association; Ex-President Georgia Medical Association; Member of the American Medical Association; Fellow of the American Association of Obstetricians and Gynecologists


### SOME CAUSES THAT LEAD TO INVALIDISM IN WOMEN.*

BY J. B. S. HOLMES, M. D., ATLANTA, GA.,

Adjunct Professor of Obstetrics and Gynecology, Southern Medical College, Atlanta, Ga.; Vice-President of the Southern Surgical and Gynecological Association; President of the Tri-State Medical Association; Ex-President Georgia Medical Association; Member of the American Medical Association; Fellow of the American Association of Obstetricians and Gynecologists.

*Gentlemen of the Tri-State Medical Association:*

It is my duty, as well as my pleasure, to thank you sincerely for the honor you have done me in selecting me to preside over your deliberations, an honor quite as unmerited as it was unexpected.

To be elevated to the presidency of the Association is a compliment that any man should be proud of, and I do not hesitate to say that I feel more flattered by it than by any of the other positions of honor which have been accorded me. I must, and do, heartily congratulate you upon the good work of your Association. Although the youngest of our Southern

*President's Address delivered to the Tri-State Medical Association at its 6th Annual Meeting, held in Atlanta, Oc[illegible], 18[illegible]4.

medical organizations, the character of its papers and discussion will compare favorably with those of any medical organization in the union. Indeed, the value of them is such that from, and including this meeting, they must be published in book form, for any physician might well be proud to have the volume upon his library shelf. I trust the members will all agree with me as to the necessity of this publication. With the increased territory that we expect to embrace—with the new and select material we hope to add, there is no reason why we should not rival, in original and investigative work, that standard of all associations, the American Medical.

I especially congratulate you upon the auspicious circumstances attending this, our sixth annual meeting. I trust your meeting, in this, the Gate City, not only of this, the Empire State, but of the South, may be the best and most productive of good to our profession of any in the history of the Association.

I have selected as the subject for my address "Some Causes That Lead to Invalidism in Women." A subject broad, comprehensive and of vital importance. There is certainly no class of our citizens who so much deserve our best efforts as "The Women of America."

I shall address my remarks principally to the family physician—the general practitioner, and especially to the young members of this class. If we, as physicians, would exert ourselves to educate the laity, especially the mothers and daughters, in the proper methods of hygiene, very much good would certainly reward our efforts; many good women would be spared years of invalidism and the happiness of many homes maintained. With a united effort on our part and a determination to accomplish it, how soon will be seen the American women that are now so rapidly deteriorating physically, begin to ascend the scale in strength and stamina? What more inviting field is there for professional ambitition? With one accord, let's resolve to put our shoulders to the wheel and never relax our efforts until the revolution is in the right direction. If we expect to have strong and healthy women, those that can withstand the storms and cyclones, through which all, even the most favored, must certainly pass, we must begin before the period of adolescence, else we fail.

Garrigues says, "Education has great influence in the development of gynecological diseases. Too great assiduity in study in early youth concentrates the nerve energy on the brain and deprives the uterus and ovaries of their share, at a time when these organs are undergoing an enormous amount of development and preparing for the important functions of womanhood and motherhood."

I endorse all he says, for I cannot condemn in language too strong the pernicious system of female education in vogue in this country. While I desire to be plainly understood as an advocate of higher education in both sexes, still, I unhesitatingly say, that the women, yes, the people of America would be infinitely better off with a strong and healthy offspring if our girls never saw anything in the school-room but a blue-back speller, an elementary grammar and the first book in arithmetic, and trust to their inclinations and tastes for reading and intellectual improvement in after years, when the stage of full maturity has been reached—than to pursue the murderous system that is now prescribed and followed in the education of our daughters. The girl of to-day is absolutely started to school before she can dress herself. The little tot is sent away at 7:30 or 8:00 in the morning, with a cold biscuit and a hard egg, and sometimes with nothing for lunch. and is expected to spend from six to eight hours in school, sitting all that time, where, in all probability, her feet cannot even touch the floor for rest and support. At ten to twelve years of age, when she should not more than know her alphabet, she is studying Latin rhetoric, English composition, mathematics. etc., to say nothing of the many hours she is obliged to spend over the piano and violin for so-called pastime and rest. I have examined the curricula of many of our female colleges and find the studies so numerous for a girl of twelve to fourteen (the time of all others when the nervous system is taxed to its utmost to stimulate and develop functions entirely dependent upon it) that there is not a man in this audience that could pursue them for a week and not be brain-tired. Yet the poor girl is expected to rise at 6 o'clock in the morning, go to the piano and practice until 7:30, then have one-half hour for breakfast and be in the school-room by 8 or half-past, where she must remain until 12:30 or 1. A short interval for dinner and she goes back and remains until 3:30 to 5 in the afternoon. In many cases she must then go to her music, remaining until at or near the supper hour. A short interval for supper and she is then either required to study for the morrow or else go back to her practicing until 9 p. m., when she is too exhausted to sleep. It will be seen that she has been forced to do more brain work in a day than the mechanic does of manual labor or than the law allows the factory operatives or the railroad engineer to do.

Legislative enactment protects the laborer, but who protects the poor brain-tired and exhausted girl? I have seen them so exhausted from brain fatigue that they could do nothing but sit and cry. This is no overdrawn statement, and certainly is it not our duty as medical men to come to their rescue? I answer, Yes, and believe every man in this Association will endorse it. I have often remonstrated with the female educators, and must say that the fault is not so much with them as with misguided, ignorant and ambitious parents. Ambitious for their daughters to graduate and come out as young ladies a year or two in advance of the daughter of their neighbor or friend. A prominent educator recently told me that he knew the system was pernicious and dangerous to the girl, but he says, "When the parents come to me with their daughters, the first question asked is, 'How soon can you graduate her?' I would say, after inquiring into her age and studies, she should take four years. I am at once met with the answer that the ——— college, with the same curriculum, would graduate her in three, and others in even two years. I must either take her and force her through or she goes to another school, where she receives the same punishment." Where such parents cannot be brought to understand the danger of such a course to their children and will persist in it, *then* it clearly becomes the duty of the law, by legislative enactment, to extend its strong arm and give protection to this class of helpless children, as well as to the strong mechanic and laborer. Indeed, the necessity for it is a thousand times greater. The parent may answer that he is financially unable to keep the daughter in school the additional two years. If he cannot make a sacrifice and do it, it is much wiser to take her away rather than sacrifice her, for if she is continued in school under such circumstances, she is sure to cost him or her husband much more in the hands of a specialist, whether he be ophthalmologist, neurologist or gynecologist, or may be all three. Most men are guided by a monetary standard, and I am not aware that the college president is an exception to the rule; therefore, if we plead for a longer period for the education of our girls, which it is clearly our duty to do, it is safe to assume that we would secure his co-operation. While to have the period of college life considerably extended would add largely to the income of the college, it would certainly, in the end, detract greatly from that of our profession, and especially from the gynecologists. The shorter the period required for forcing a girl through school, the more she must and will suffer from it, and the surer she will fall into our hands. I am happy to say that the medical profession, in its devotion to the welfare and preservation of those entrusted to it for protection and care, rises far above any mercenary thought when dealing with those we are in honor bound to protect. Any member of our profession who, for dollars and cents, should ever lend encouragement to such a cursed course, should have his diploma taken from him and be branded as a traitor. Does any intelligent physician believe that it is right for a girl, either just verging upon menstruation, or one upon whom nature is making a hard and desperate fight to maintain this function, should be confined in a miserably ventilated and imperfectly-heated school-room, in indeed one of any kind, for eight to twelve hours daily? Shall she use her brain as long as a mechanic does his muscle? With music and a dozen or more studies, and almost as many teachers, each anxious for their pupil to be more proficient in their own department, driving and urging the poor girl on to such an extent that it is a wonder so many survive. Classes are frequently crowded, and it is not an unusual occurrence for the entire school hours to be taken up in recitations, thus causing the girl to devote her recesses to study in preparation for the next lesson. Added to this, she is in every conceivable way rushed forward. Her pride is worked upon to the highest degree, prizes are offered and marks of merit given for extra work. The disgrace, so-called, for non-promotion is impressed with due emphasis. Cramming for the examinations in the presence of the commissioners or trustees, and, worst of all, the forcing and overwork necessary for the show and display of public commencements, which I think should be entirely done away with, as tending, by the strain, overwork and emotion exercised upon the girl to do a vast and perhaps irreparable amount of harm to her nervous system. After ten months of hard study and confinement she is certainly in no condition to bear this additional strain. So high and eminent authority on nervous diseases as Dr. Weir Mitchell says: "I firmly believe, and I am not alone in this opinion, that as concerns the physical future of our women, they would do far better were they more lightly tasked and the school hours but three or four a day until they reached at least the age of seventeen. Anything, indeed, would be better than the loss of health, and if it be a question of doubt, the school, unhesitatingly, should be abandoned or its hours greatly lessened, as at least in part the source of very many of the nervous maladies with which our women are troubled." "I am almost ashamed," he says, "to defend a position which is held by many competent physicians, and if an intelligent friend asks me why it is that overwork of the brain should be so serious an evil to women at the age of womanly development, my best answer would be: 'The experience and opinions of those of us who are called upon to see how many school-girls are suffering in health from confinement, want of exercise and from brain work at a time when the nervous system is so sensitive and irritable, and at no other time is an abundant supply of fresh air and exercise so important.'" The same authority says: "It were better not to educate girls at all between the ages of fourteen and eighteen, unless it can be done with careful reference to their bodily health. To-day the American woman is, to speak plainly, too often unfit physically for her duties as woman, and is, perhaps, of all civilized females, the least qualified to undertake those weightier tasks which tax the nervous systems of women. She is not fairly up to what nature asks of her as wife or mother. How few mothers are there to-day, in the higher educated class of women, who have been rushed through college and played the devotee to fashion and society that can nurse their offspring!"

If any one doubts the above assertion, and especially the last clause, let him refer to the medical journals and read the numerous advertisements of baby foods. I venture the assertion that there are one half-dozen, or may be more, brands exhibited in the building to-day, a God-send to the little motherless (so to speak) infants. Nor is this all. Many of the mothers are so weak that they are physically unable to carry the fœtus to full term, or if they should, it is so weak that to save it, it must be placed in an artificial mother—an incubator. See the contrast in our strong country girls, raised to outdoor work and accustomed to outdoor exercise. In the higher classes you will see many beautiful faces, but figures entirely too spare; in many cases with a waist constricted to an extent that amounts to a deformity. While the faces of many of them are beautiful to look at, out of say fifty that you would chance to meet, how many are fit for wives and mothers, with weak backs, neuralgia and the various manifestations of hysteria, that domestic terror that has brought untold discomfort to many homes. The destiny of many of these weak and nervous little creatures is either the lounge, the resorts or homes for invalids. Many, yes, very many, seek relief from their suffering by resorting to whiskey, opium, cocaine and like narcotics. There is not a physician in this audience but that can recall among his acquaintances one, or perhaps many, highly-educated women, surrounded by every comfort, who are victims to one or the other of the above mentioned vices. Only the physician and husband can tell what one of these invalids can do to make a home wretched.

Dr. Cyrus Edson very truthfully says, "At the present moment, there is in this country a condition existing among the women, which is the cause of the gravest alarm so far as I can learn. First, from that personal knowledge that comes to each physician in his practice, and second from the concensus of knowledge had by all physicians and reflected in the medical magazines and journals, the problem before the medical men of to-day is 'The health of the educated woman,' of those women who represent three or four generations born on American soil. While it is true, from time to time, articles appear in the lay publications in which this problem is alluded to, it is only in the medical publications that the evil is thoroughly reflected. None know better than I do the difficulty which makes itself felt when we would discuss the subject. Modern canons of taste forbid any but the most distant allusions to what is going on among us in many ways, and he is considered immodest, or even indecent, who seeks to draw the veil. At the same time things have come to such a point that our progress as a nation may be seriously impaired unless we can regulate the evil and find a remedy. Were this not true the aggregate of torture inflicted upon the American women would, in itself, be enough to make any one speak out, who realizes what it is. There are probably sixty thousand women in the United States who suffer month in and month out, who have suffered for years and who are destined to for years to come. Is not this cause for speech? More than this, there are probably sixty-five thousand girls now growing up who will suffer as their mothers have and do suffer. It seems to me that when we realize such a condition of things we should feel the necessity of its being seriously and candidly considered. Let us briefly state facts as they are.

"An American girl educated as it is our pride to educate her, marries the man of her choice amid the warm congratulations and good wishes of all her friends. She is clever, bright, beautiful and looks forward to years of happiness and usefulness. One, or at most, two children are born, and if we meet her, we can scarcely recognize her. She looks haggard and worn, she is fretful and peevish, and has become a burden to her husband instead of a help to him. She feels as if she were a nuisance to herself and others. Worse than all, she is a confirmed invalid, doomed to suffer more or less during the coming years, and these alas, may be many. Expressed in the fewest words, the evil is that an increasing large proportion of the women of the American race are unable to perform their functions as mothers, and these women, include, mentally, the best we have amongst us." Right here allow me to offer a few suggestions for the relief, or rather the prevention of the evils alluded to. Up to the age of ten or twelve, or until we have every evidence of approaching menstruation, the girl should be treated as a boy. In referring to the approach of menstruation, I must not fail to speak of the irreparable damage that the young girl, entirely ignorant of the menstrual functions, does herself when the flow first appears. She feels that something has occurred to disgrace her and makes every possible effort to suppress it; bathes in cold water, uses ices, etc.—will do anything to stop it. A sudden pelvic congestion, with its inflammatory sequence, leaving permanent invalidism, results in many cases. Many of you are familiar with instances of this kind; where the ill health of women is clearly attributable to this cause. It is clearly and manifestly the duty of the mother to carefully inform the daughter of this function. She should be carefully instructed as to its expected approach and as to the care required before and during the period. A mother who fails to do this is culpable to the highest degree, and should the innocent girl, from want of knowledge, which it is the duty of the mother to impart, become an invalid from this cause, the mother should always feel that she is responsible for her child's suffering. It is truly astonishing to know the number of apparently sensible women, who, from a sort of false modesty, fail to give their daughters this important information.

To return to the girl's physical training—Up to the age of adolescence she should be treated as a boy, should be taught to engage in outdoor sports that tend to develop the muscular and nervous systems. Teach them to run, climb, play tennis, ride and drive, shoot, fence, etc., in fact, make "Tomboys" so-called, out of them and not hot-house plants.

Teach them house-keeping, etc., and as the time approaches for menstruation the more violent forms of exercise should cease. The girl will be able all the better to bear this changed condition after the thorough physical training she has had. With her strong constitution she has nerve strength sufficient to meet the change, and really should hardly know, from any discomfort, that it has occurred. When her menstruation occurs she should be taught to rest as much as possible during the continuance of the flow. Where it is practicable, she should be kept in bed (during menstruation) for the first three or four, at least for the first few years of her menstrual life. She will be much better for such enforced quietude and freedom from the wear and tear of her nervous energy incident to youthfulness, at a time when her system is learning to accommodate itself to a new condition of things. During these days of rest make it pleasant for her. She will soon be educated to this monthly quietude and accept it without complaint. Moreover, an impression is firmly made upon her in childhood of the necessity for care during menstruation, a lesson she is apt to profit by in future years. Unless she is so reared and educated as to understand the full importance of this, an otherwise strong and healthy girl may have her life wrecked by imprudence during menstruation. She pays little or no attention to her condition, dons the evening dress, removing perhaps heavy clothing, skates, goes to theatres and germans, dances immoderately in a close room (heated often to a high temperature), and while greatly heated, she promenades on the veranda in the night air or drives home at three in the morning without wraps, and that too at a time when a process is going on that can be so easily strained in an abnormal direction. From such carelessness, or ignorance, menstrual disorders or pelvic disease, often of serious character, result. I can now recall a case of a beautiful young woman who replaced her winter clothing by a light evening dress, attended the german, dancing until 2 a. m., and drove home without wraps while menstruating. I saw her twelve hours after in a severe chill, menstruation stopped, great pelvic congestion, resulting in acute ovaritis and pelvic peritonitis. To-day she has a fixed ovary and her periods are looked forward to with fear and dread on account of the pain suffered at that time.

I have seen several similar cases, and dare say many of my hearers have had a like experience. Where it is impossible to give the girl complete rest during the first few years of her menstrual life, lighten her duties as much as possible. If necessary, legislative enactment should cause seats to be placed in the stores for shop girls when not actually employed. It is well known that the nervous system presides over, regulates and directs, so to speak, the functions of the different organs. For menstruation to be maintained the nervous system must be strong and healthy. When this is weak we must expect either suppressed, painful or irregular menstruation; hence the great and absolutely imperative necessity in childhood of looking carefully to the upbuilding of a strong constitution—one that has sufficient nerve force to meet these demands. Menstruation must be regarded as a nervous or reflex act, and while this is true, a girl must, of course, have properly formed genital organs, with a sufficiency of blood for the function to be kept normal. Though, with these requirements met, amenorrhœa is quickly produced from overtaxed brain, as I have often seen among school-girls. They return to school in September or October, after a two or three months' vacation, rested mentally. Ninety-five per cent. will probably give a history of regular menstruation, at least as to time. The following spring, just before the commencement, after ten months of so-called educational training, not ten per cent. will be free from some menstrual disorder; in the greater number total suppression will exist. This is not guess work, but actual observation and experience. Ten or twelve years of age is early enough to start a girl to school. Give her not more than two or three hours of light study in the forenoon, and not more than two in the afternoon, allowing at least two hours of rest at noon; and during this time she should be required to take a lunch, and not do as many girls in cities do, wait until school dismisses, in the afternoon, before taking food, which is so essential to supply the waste and wear constantly going on. It should also be seen that she takes breakfast before leaving home in the morning. It should always be understood with the faculty that her brain work is to be greatly lessened or even suspended during menstruation. Nothing can be more pernicious than the present mode of teaching. The studies should be but few, and the mental taxation very light up to seventeen. Up to that age a girl should never be taxed mentally, for the nerve force so necessary to her development is perverted and expended in mental work, resulting in delay or imperfectly developed genital organs, the last developed and the first to fail in performing their functions. Not only up to seventeen should mental work be light, but regular exercise in the open air required. A neglect of this is one of the potent factors in the delicate constitutions in city-raised girls. All school buildings for girls should be provided with elevators. Going up and down stairs many times a day is certainly very injurious, fatiguing and productive of great harm. Music should not be taken until the girl is sixteen or seventeen years of age, when her nervous system has become thoroughly accustomed to the menstrual function, and strong enough to bear the tax and strain. While music is regarded by the parents and most teachers as a rest and recreation from study, it is really, on account of its effect on the emotions, the hardest and most trying on the nervous system in the college curriculum, and under no circumstances should the school-girl be allowed to spend her recess hours (those intended for rest and diversion) in music study and practice.

(TO BE CONTINUED.)

